# Downstaging therapy of intermediate-stage hepatocellular carcinoma: microwave ablation vs. surgical resection

**DOI:** 10.3389/fonc.2026.1868497

**Published:** 2026-08-05

**Authors:** Gang Li, Xiaogang Hu, Hongyu Wang, Wendao Liu

**Affiliations:** 1 Department of Oncology and Vascular Intervention, Affiliated Jinhua Hospital, Zhejiang University School of Medicine, Jinhua, Zhejiang, China; 2 Department of Interventional therapy, Guangdong Provincial Hospital of Chinese Medicine and Guangdong Provincial Academy of Chinese Medical Sciences, Guangzhou, Guangdong, China

**Keywords:** conversion therapy, hepatocellular carcinoma, microwave ablation, propensity score matching, surgical resection

## Abstract

**Background:**

Transarterial chemoembolization(TACE) is a potential downstaging therapeutic strategy for intermediate-stage hepatocellular carcinoma(HCC). However, therapeutic options following conversion therapy is still controversial.

**Purpose:**

This study aimed to compare the efficacy and safety of surgical resection(SR) and microwave ablation(MWA) following TACE based downstaging therapy for intermediate-stage HCC.

**Methods:**

From January 2008 to December 2021, a total of 2,125 consecutive patients with intermediate-stage HCC underwent initial TACE at seven hospitals were identified. Among them, 554 eligible HCC patients occurred objective response(OR) to TACE, who meet the tumor burden equivalence of early-stage disease. The propensity score matching(PSM) was applied to reduce selection bias. The overall survival(OS) and recurrence-free survival (RFS) were compared using the Kaplan–Meier method with the log-rank test. Independent risk factors for survival outcomes were identified through multivariable Cox analyses using forward stepwise regression.

**Results:**

After PSM 1:1, 152 patients in MWA group were matched with those in SR group. SR demonstrated comparable long-term survival outcomes (5-years OS rate, 85.3% vs 82.5% and 5-years RFS rate, 77.2% vs 75.0%) than MWA. Multivariate analyses showed that the factors that significantly affected the OS were Body Mass Index (hazard ratio[HR]:0.61;95% confidence interval [CI]: 0.37-0.82). Multivariate analyses showed that the interval between local-region treatment and TACE (HR:0.41; 95% CI: 0.24-0.67), HBV (HR: 0.32; 95% CI: 0.15-0.69) and systemic treatment (HR:0.44; 95% CI: 0.25-0.76) significantly affected the RFS.

**Conclusions:**

MWA might be acceptable as an alternative to SR in first-line TACE based downstaging therapeutic scheme for patients with intermediate-stage HCC.

## Introduction

Over the past two decades, the Milan criteria have been widely recognized as the gold standard for selecting candidates with hepatocellular carcinoma (HCC) who are suitable for liver transplantation (LT) (1–3). Nevertheless, the limited availability of donor organs restricts the widespread clinical application of this therapeutic approach. Surgical resection (SR) and thermal ablation (TA) have emerged as alternative curative options for patients who are not suitable for LT (4, 5). Regrettably, over 70% of HCC patients receive their initial diagnosis during intermediate or advanced stages, thereby missing the chance for SR or TA. In light of this, the National Comprehensive Cancer Network (NCCN) Guidelines suggest that transarterial or systemic therapies, such as transarterial chemoembolization (TACE) or the combination of atezolizumab and bevacizumab, should be considered for unresectable HCC (3, 6, 7).

Serving as the standard first-line therapy for intermediate-stage HCC, TACE has been reported to achieve an objective response rate (ORR) of 30-50% in previous studies (8, 9). Consequently, a subset of patients with intermediate-stage tumor burden may convert to an early-stage tumor burden after TACE, as confirmed by further imaging assessments—this process is clinically defined as downstaging. To achieve a curative outcome, additional local regional treatment is often administered following TACE. A series of studies have demonstrated that HCC patients who initially met the BCLC A stage and those who achieved downstaging via TACE exhibit comparable survival outcomes. For instance, Shi et al. reported that microwave ablation (MWA) yields similar results between patients initially meeting the Milan criteria and those receiving MWA following TACE-induced downstaging for tumors originally beyond the Milan criteria (10).

To date, Yuan et al. have illustrated that SR offers a survival advantage over the “watch-and-wait” approach in HCC patients who achieve an objective response (OR) to TACE and meet resectability criteria (11). Despite these promising findings, evidence remains limited concerning the optimal choice between SR and MWA following successful conversion therapy for initially unresectable HCC (uHCC). Among all ablation methods, MWA proves particularly suitable for this application, with mang advantages, such as more rapid intratumoral temperature elevation (12–14). Previous studies, including our own, has demonstrated that MWA and SR yield comparable survival outcomes and safety profiles when utilized as upfront treatments for 3–5 cm HCC, and both modalities demonstrate comparable durable efficacy when employed as subsequent treatments following downstaging therapy (15, 16).

Despite the encouraging results obtained so far, treatment options for uHCC after downstaging remain controversial. And the evidence remains inadequate to direct the choice between SR and MWA after downstaging therapy for intermediate-stage HCC, Therefore, this multi-center study sought to examine and contrast the long-term survival outcomes of SR and MWA in individuals with initially intermediate-stage HCC who attained successful TACE-based downstaging.

## Materials and methods

This investigation was executed per the principles of the 1975 Declaration of Helsinki and was sanctioned by the Institutional Review Board (IRB) of Guangdong Provincial Hospital of Chinese Medicine (BE2023-343-01). Written informed consent was not required given the retrospective study design.

### Patients

When the radiological diagnostic criteria for HCC established by the American Association for the Study of Liver Diseases (AASLD) remained unmet (3), biopsy was advised before TACE. This retrospective, multi-center investigation encompassed 2,125 consecutive patients with intermediate-stage HCC (as per the Barcelona Clinic Liver Cancer [BCLC] guideline) who received initial TACE from January 2008 through December 2021. Follow-up data were censored on October 31, 2024.

### Noninvasive evaluation criteria of downstaging

Downstaging evaluation was executed by two fellowship-trained abdominal radiologists at each participating hospital (with 5–10 years of experience in liver imaging) using the mRECIST criteria (17). Based on contrast-enhanced computed tomography (CT) or magnetic resonance imaging (MRI), a complete response (CR) was characterized as no enhancement in the arterial phase of target lesions, whereas a partial response (PR) was characterized as a decrease of at least 30% in the total diameter of the active portion of visible lesions (enhancement in the arterial phase). The objective response (OR) was characterized as the sum of CR and PR. A significant decrease in alpha-fetoprotein (AFP) levels was also considered an important reference standard. Successful downstaging was defined as the conversion of intermediate-stage HCC to early-stage HCC (as per the BCLC guideline) following an OR to TACE, which requires the number of visible lesions to be reduced to a single lesion or 2–3 lesions with a sum of maximum tumor diameters < 3 cm, without macrovascular invasion or extrahepatic metastasis.

### Study design

A multidisciplinary team consisting of hepatologists, surgeons, oncologists, and radiologists recommended curative-intent downstaging treatment for a series of HCC patients who achieved an OR to TACE, in accordance with the criteria outlined in **Supplementary Information E1.1**. The downstaging cohort included patients who initially met BCLC stage A and underwent SR or MWA as first-line treatment. Detailed procedures for TACE, protocol discontinuation, SR, and MWA are outlined in **Supplementary Information E1.2-1.5**. The technical efficacy of SR and MWA was evaluated using multi-phase imaging performed 1 month after treatment. For SR, an R0 resection was required, while for MWA, a 5 mm safe margin was achieved after a maximum of two treatment sessions.

**Figure 1:**
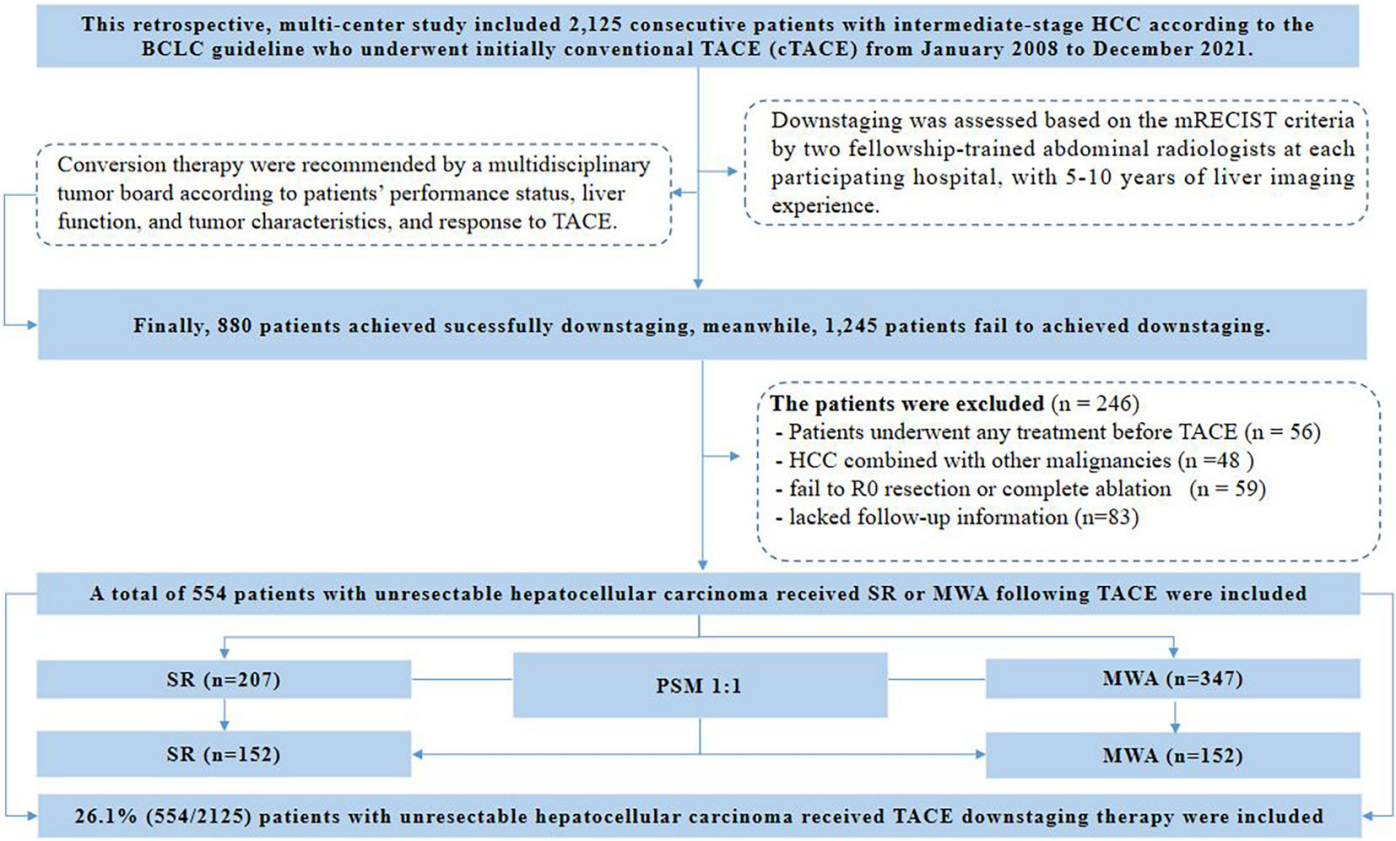
Enrollment pathway of patients with unresectable hepatocellular carcinoma (HCC) who underwent successful transarterial chemoembolization (TACE) downstaging therapy. Note. - Successful downstaging therapy was defined as viable intrahepatic tumor burden met the Milan criteria (one lesion up to 5 cm or no more than three lesions ≤3 cm without vascular invasion or extrahepatic metastasis). HCC, hepatocellular carcinoma; TACE, transarterial chemoembolization; SR, surgical resection; MWA, microwave ablation; PSM, propensity score match.

Ultimately, 880 patients achieved successful downstaging, while 1,245 patients failed to do so. The inclusion criteria following downstaging were: (a) age ranging from 18 to 80 years; (b) Eastern Cooperative Oncology Group (ECOG) performance status < 2; (c) Child-Pugh class A liver function; (d) OR to TACE; (e) baseline BCLC stage A after TACE; (f) lesions eligible for SR or MWA, as verified by a panel of liver experts. Patients were excluded if they: (a) had BCLC stage B; (b) had undergone previous antitumor treatment prior to downstaging therapy; (c) demonstrated current or past malignancies aside from HCC; (d) did not achieve R0 resection or complete ablation; (e) lacked follow-up information. The enrollment pathway is illustrated in **Figure 1**. In total, 554 patients met the inclusion and exclusion criteria, with 207 allocated to the SR group and 347 to the MWA group.

### Follow-up protocol

**Figure 2:**
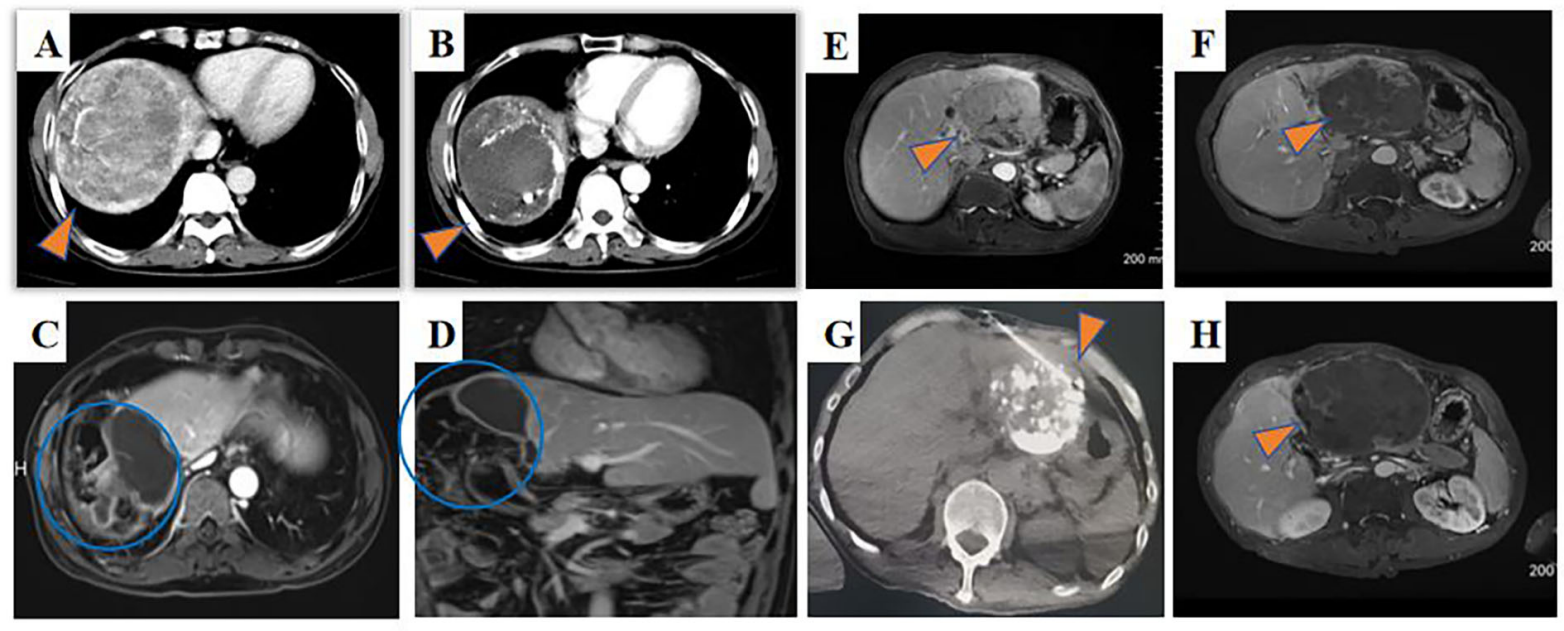
The imageological medical record of uHCC patient underwent the surgical resection (SR) after transarterial chemoembolization (TACE) downstaging therapy. A 58-year-old man with large hepatocellular carcinoma surrounding satellite lesions (11.2 × 10.9 × 10.8 cm) with received TACE combined with sequential SR. **(A)** Arterial phase CT shows a high density with a complete pseudocapsule (orange arrow); **(B)** After three cycles of TACE, an area necrosis occurs inside the tumor and tumors deposit iodine oil well in the arterial phase (orange arrow) and the AFP level was 18.2 ng/ml; **(C, D)** SR of the target tumor under laparoscopy, and axial and sagittal views show partial resection of the liver with tumor (blue cycle). The imageological medical record of uHCC patient underwent the microwave ablation (MWA) after transarterial chemoembolization (TACE) downstaging therapy. A 48-year-old man with large hepatocellular carcinoma (9.2 ×8.9 × 8.5 cm) received TACE combined with sequential ablation. **(E)** Enhanced contrast CT (arterial phase) shows a massive tumor with rich blood supply (orange arrow) and the AFP level was >121000 ng/ml; **(F)** After three cycles of TACE, an area necrosis occurs inside the tumor in the arterial phase (orange arrow) and the AFP level was 105.3 ng/ml; **(G)** CT guided MWA for active regions involved in the margin of large tumors **(H)** The residual lesion disappear completely followed by CT after MWA.

Follow-up medical records for patients who converted from TACE to SR or MWA are shown in **Figure 2**. Routine contrast-enhanced imaging and serum tumor markers (e.g., AFP and des-gamma-carboxy prothrombin [DCP]) were evaluated every 4–6 weeks in the first year after initial SR or MWA, and every 3 months thereafter. If suspected distant metastasis was detected, chest CT, whole-body bone scans, or positron emission tomography (PET)-CT were selectively executed. If recurrent lesions were identified, corresponding interventional therapy was administered, and detailed information is illustrated in **Supplementary Table 1**. Sixteen clinicopathologic variables before TACE were collected, and these are demonstrated in Supplementary Information E1.6.

### Outcomes definitions

Overall survival (OS): The interval from the first TACE procedure to death from any cause or last follow-up.

Recurrence-free survival (RFS): The interval from downstaging therapy initiation to initial documented tumor recurrence (definitively identified through radiological and/or histological criteria as HCC, tumor-in-vein, or distant metastasis, per the AASLD) or death from any cause.

Aggressive recurrence-free survival (ARFS): The timeframe from downstaging therapy initiation until initial tumor recurrence that surpasses the Milan criteria.

Advanced-stage recurrence-free survival (ASRFS): The time interval from downstaging therapy initiation to the initial occurrence of cancer thrombus or distant metastasis.

### Statistical analysis

Quantitative variables were denoted as mean ± standard deviation or median with interquartile range (IQR) and analyzed employing the Kruskal-Wallis test. Qualitative variables, shown as frequencies, were examined using the chi-square test. After successful downstaging, patient characteristics in the SR group were contrasted with those in the MWA group. Propensity score matching (PSM) with a nearest-neighbor algorithm (1:1) was employed to reduce confounding variables between the two treatment groups (13). Propensity scores derived from the PSM procedure were subsequently utilized for case-weight estimation (inverse probability treatment weighting, IPTW) (18). Weights for patients undergoing SR represented the inverse of the propensity score, whereas weights for patients undergoing MWA represented the inverse of 1 minus the propensity score. Kaplan-Meier survival curves were utilized to estimate cumulative survival rates, with differences assessed employing the log-rank test. Independent prognostic factors for survival outcomes were determined through univariate and multivariable analyses employing the forward stepwise Cox regression model.

Statistical analyses were executed utilizing SPSS version 26.0 (IBM Corp., NY, USA) and the RMS package within R software version 3.6.4 (http://www.r-project.org/). All statistical tests employed two-sided approaches, with P-values < 0.05 deemed statistically significant.

## Results

### Downstaging condition

Among the 2,125 patients with initially intermediate-stage HCC who received downstaging therapy, 554 (26.1%) achieved successful downstaging. The median number of TACE sessions among these 554 patients was 2 (range, 1–6 sessions). Additionally, the median interval between TACE and SR was 52 days (range, 21–76 days), while the median interval between TACE and MWA was 58 days (range, 37–85 days). Baseline characteristics of the cohorts categorized by downstaging therapy are presented in **Supplementary Table 1**. The mean maximum tumor diameter reduced from 7.6 ± 1.1 cm to 4.2 ± 0.5 cm after multiple cycles of TACE. Patients in the downstaging therapy group exhibited significantly higher survival times, encompassing OS, RFS, ARFS, and ASRFS, relative to those in the non-downstaging therapy group (all, *P* < 0.001; **Supplementary Figure 1**).

### Baseline characteristics

**Table 1** presents baseline characteristics of both unadjusted and adjusted cohorts categorized by the two treatment approaches. Prior to 1:1 PSM, the MWA group demonstrated a markedly elevated mean age (56.2 ± 9.7 years) relative to the SR group (52.8 ± 10.2 years) (*p* = 0.024). The percentage of patients with ECOG = 1 was notably reduced in the SR group (1.0%, 2/207) versus the MWA group (4.0%, 14/347) (*p* = 0.026). After 1:1 PSM, 152 patients were allocated to both the SR and MWA groups, respectively. Baseline characteristics of participants were balanced through the nearest-neighbor method using a 0.1 caliper, with all adjusted variables reaching optimal balance (**Supplementary Figure 2**).

**Table 1:** Baseline Characteristics of the patients with HCC who received downstaging treatment.

Variables	Unmatched		P-value	PSM (1:1)		P-value
	SR group (n, 207)	MWA group (n, 347)		SR group (n, 152)	MWA group (n, 152)	
Age ± SD (years)	52.8 ± 10.2	56.2 ± 9.7	0.024*	50.8± 10.4	49.6 ± 10.0	0.378
BMI ± SD (kg/m2)	23.1 ± 4.5	23.3 ± 4.2	0.879	22.3± 4.4	22.3± 4.0	0.987
Sex			0.482			1.000
Female	28 (13.5)	50 (14.4)		19 (12.50)	19 (12.50)	
Male	179 (86.5)	297 (85.6)		133 (87.50)	133 (87.50)	
ECOG			0.026*			0.126
0	205 (99.0)	333(96.0)		151 (99.34)	146 (96.05)	
1	2 (1.0)	14(0.4)		1 (0.66)	6 (3.95)	
Smoking			0.537			0.113
Absence	145(70.0)	253(72.9)		123 (80.92)	134 (88.16)	
Presence	62(30.0)	94(27.1)		29 (19.08)	18 (11.84)	
Drinking			0.085			0.873
Absence	171(11.5)	314 (3.4)		130 (85.53)	128 (84.21)	
Presence	36(88.5)	33 (96.6)		22 (14.47)	24 (15.79)	
Comorbidity			0.537			0.377
Absence	176(11.5)	301(89.7)		8 (5.26)	4 (2.63)	
Presence	31(88.5)	46(10.3)		144 (94.74)	148 (97.37)	
HBV			0.085			1.000
Absence	8(11.5)	24(3.4)		151 (99.34)	151 (99.34)	
Presence	199(88.5)	323(96.6)		1 (0.66)	1 (0.66)	
HCC_number at TACE			0.029*			1.000
4-6	134(43.8)	15(25.9)		96 (63.16)	97 (63.82)	
>6	73(56.2)	43(74.1)		56 (36.84)	55 (36.18)	
HCC_diameter at TACE			0.684			0.550
≤ 7cm	148(51.7)	285(48.3)		61 (40.13)	52 (34.21)	
> 7cm	59(48.3)	62 (51.7)		80 (52.63)	89 (58.55)	
ALBI grade at TACE			0.767	11 (7.24)	11 (7.24)	
1	131(71.3)	207 (69.0)				0.598
2	76 (28.7)	140 (31.0)		99 (65.13)	97 (63.82)	
AFP at TACE			0.210	53 (34.87)	55 (39.18)	
≤ 400 ng/ml	160(26.4)	262(36.2)				0.676
> 400 ng/ml	47(73.6)	85(63.8)		117 (76.97)	121 (79.61)	
AST (U/L), median, IQR	92 (34, 158)	96 (42, 202)	0.885	35 (23.03)	31 (20.39)	
ALT (U/L), median, IQR	65 (22, 98)	68 (26, 85)	0.714	65.00 (34.63-86.00)	51.90 (33.48-74.50)	0.581
TBIL(μmol/L), median, IQR	18.4 (10.2, 21.5)	17.1 (9.9, 22.7)	0.440	46.00 (34.00-67.20)	45.00 (36.85-60.80)	0.158
ALB (g/L), mean ± SD	41.9 ± 5.3	40.3 ± 7.1	0.548	14.05 (11.20-19.55)	12.50 (9.75-18.83)	0.300
INR, mean ± SD	1.09 ± 0.12	1.11 ± 0.10	0.721	1.05 (1.02-1.10)	1.09 (1.00-1.16)	0.224
PT (s), mean ± SD	12.8 ± 0.8	12.8 ± 0.6	0.947	12.10 (11.70-12.70)	12.50 (11.50-12.70)	0.176
PLT (×109), median, IQR	178 (102, 215)	153 (114, 224)	0.772	160.00 (127.18-203.50)	150.00 (92.10-203.75)	0.064
CRP (U/L), median, IQR	12.9 (4.5, 19.8)	10.7 (4.2, 15.5)	0.427	2.63 (1.20-23.59)	3.15 (1.20-23.59)	0.552
Neutrophil (μmol/L), mean ± SD	4.3 ± 0.5	3.9 ± 0.5	0.627	4.52 (3.31-4.70)	4.52 (3.00-4.70)	0.192
Lymphocytey (μmol/L), mean ± SD	1.8 ± 0.2	1.7 ± 0.3	0.882	1.60 (1.50-2.00)	1.60 (1.60-1.98)	0.805
The median interval (days)			0.210			0.925
≤ 60	131(63.3)	207(59.7)		110 (72.37)	112 (73.68)	
> 60	76 (36.7)	140(40.3)		42 (27.63)	40(26.32)	
Systemic treatment			<0.001			0.637
Absence	145 (70.05)	290 (83.57)		126 (82.89)	130 (85.53)	
Presence	62 (29.95)	57 (16.43)		26 (17.11)	22 (14.47)	

Unless otherwise indicated, data are number of patients, and data in parentheses are percentages.

* P-value < 0.05. SR, surgical resection; MWA, microwave ablation; PSM: propensity score match; HCC, hepatocellular carcinoma; BMI, body mass index; ECOG, Eastern Cooperative Oncology Group; HBV, hepatitis type B viral; AFP, α-fetoprotein; ALBI, albumin-bilirubin; ALB, albumin; ALT, alanine aminotransferase; AST, aspartate aminotransferase; PT, prothrombin time; INR, international normalized ratio; TBIL, total bilirubin; PLT, platelet; CRP, C-reactive protein.

### Comparison of long-term survival outcomes before PSM

**Figure 3:**
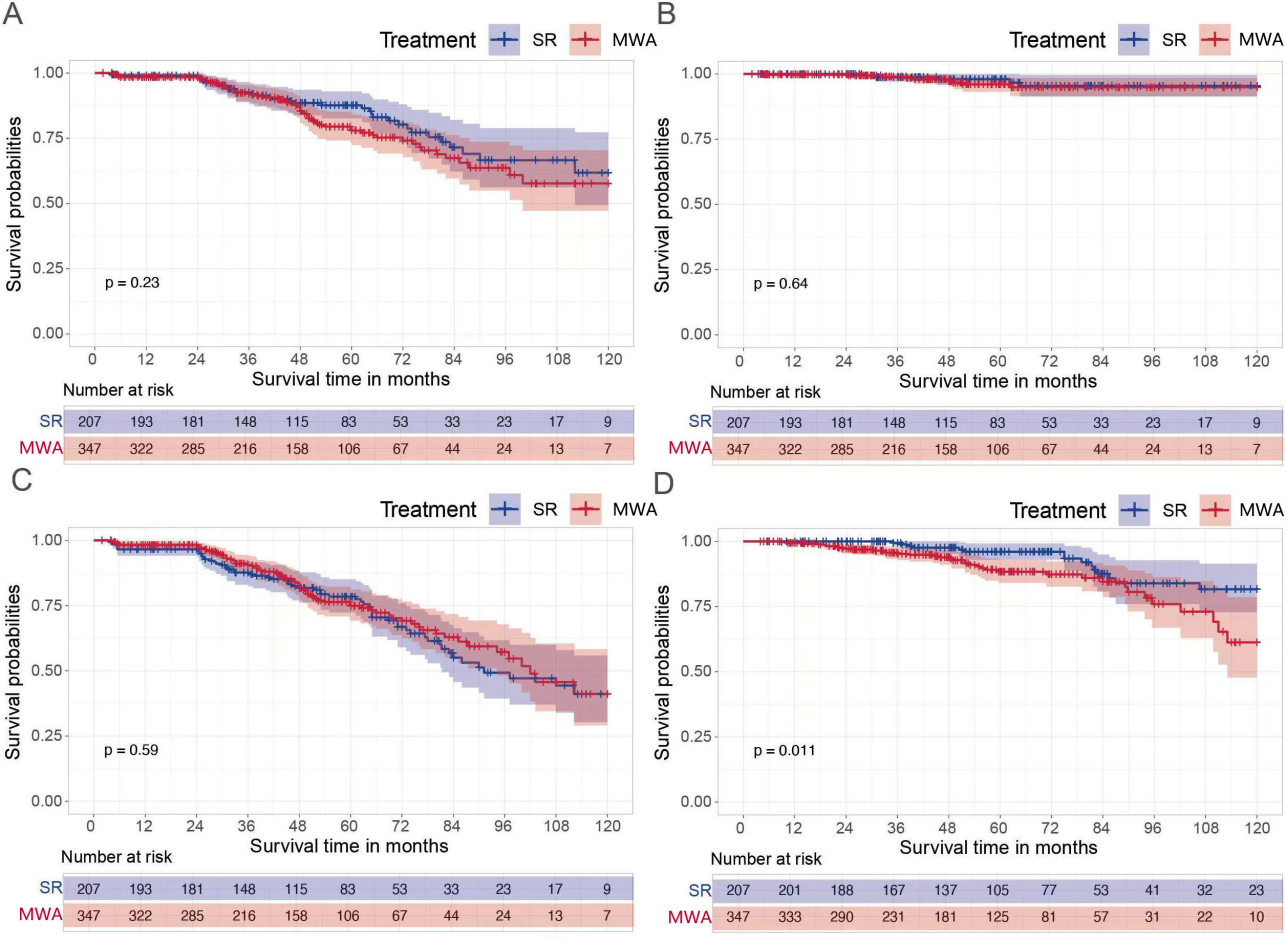
Comparing the survival of SR and MWA groups for uHCC patients in downstaging cohorts before PSM. Kaplan–Meier curves for the **(A)** aggressive recurrence-free survival (ARFS) and **(B)** advanced-stage recurrence-free survival (ASRFS), **(C)** recurrence-free survival (RFS) and **(D)** overall survival (OS) of uHCC patients between SR group and MWA group before propensity score matching (PSM). Note. - The variables matched for PSM included age at diagnosis, gender, ECOG, comorbidity, HBV, ascites, HCC number, HCC diameter, ALBI grade, AFP, tyrosine kinase inhibitors and immunotherapy. uHCC, unresectable hepatocellular carcinoma; SR, surgical resection; MWA, microwave ablation; PSM, propensity score match.

The median follow-up period was 67.4 months (IQR: 45.2–91.6 months) for the SR group and 71.8 months (IQR: 52.5–91.2 months) for the MWA group. Crude Kaplan-Meier analyses displayed no significant differences in cumulative ARFS rates between both groups: 97.5%, 88.2%, 84.5%, and 61.7% at 1, 3, 5, and 10 years in the SR group, relative to 96.8%, 87.8%, 76.5%, and 58.3% in the MWA group (**Figure 3A**, p = 0.233). Similarly, no significant differences were observed in cumulative ASRFS rates: 99.1%, 99.1%, 98.8%, and 97.7% in the SR group versus 98.9%, 97.8%, 96.5%, and 94.3% in the MWA group at the same time points (**Figure 3B**, p = 0.643). Cumulative RFS rates were also comparable between the two groups: 98.2%, 92.8%, 78.5%, and 37.9% in the SR group versus 96.6%, 90.3%, 75.2%, and 37.7% in the MWA group (**Figure 3C**, p = 0.592). The cumulative OS rates were higher significantly in the SR group (98.5%, 93.2%, 90.5%, and 77.7% at 1, 3, 5, and 10 years) versus those in the MWA group (98.3%, 90.8%, 86.5%, and 64.3%) (**Figure 3D**, p = 0.011).

### Comparison of long-term survival outcomes after PSM

**Figure 4:**
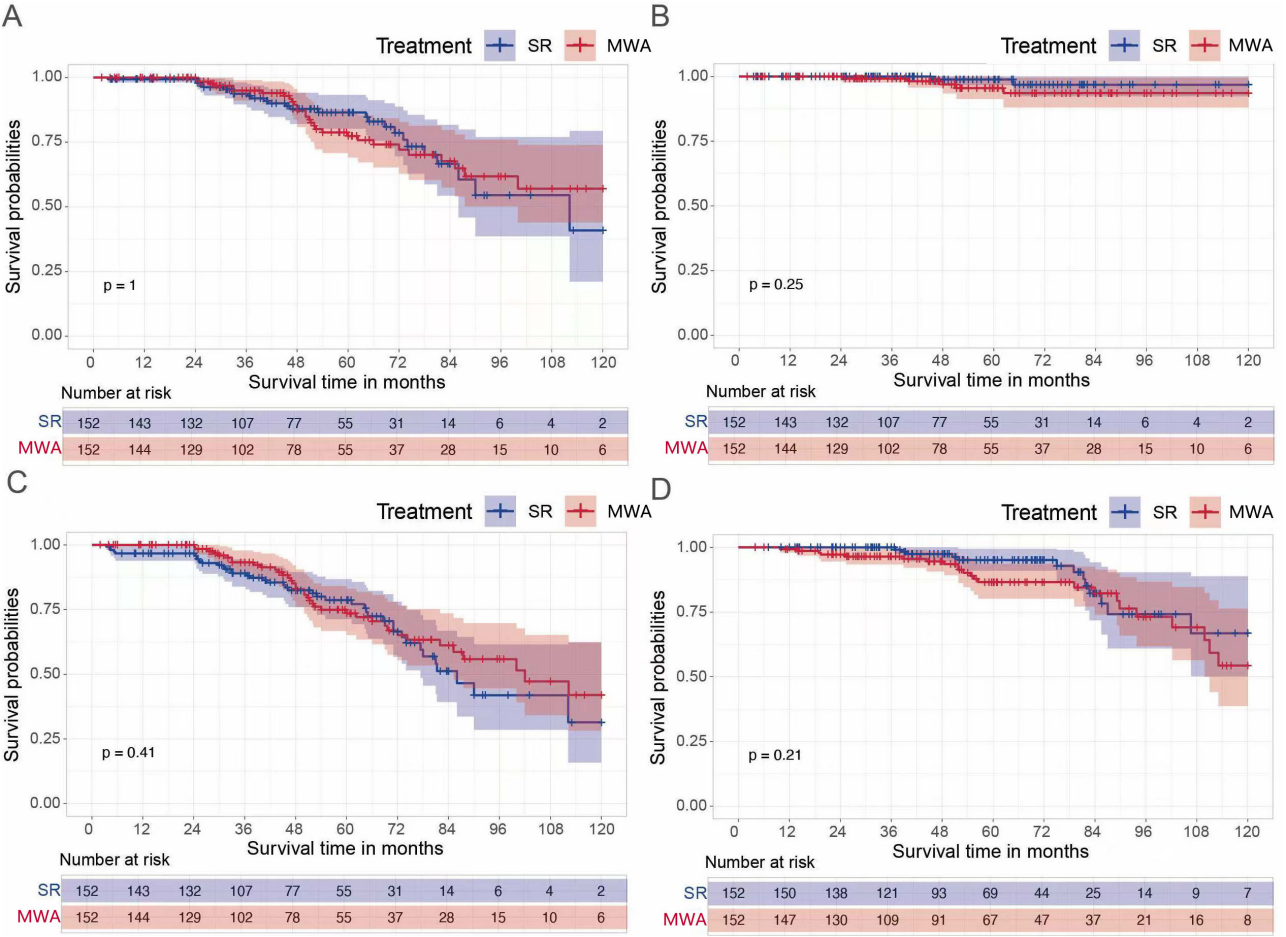
Comparing the survival of SR and MWA groups for HCC patients in downstaging cohorts after PSM. Kaplan–Meier curves for the **(A)** aggressive recurrence-free survival (ARFS) and **(B)** advanced-stage recurrence-free survival (ASRFS), **(C)** recurrence-free survival (RFS) and **(D)** overall survival (OS) of uHCC patients between SR group and MWA group after propensity score matching (PSM). Note. - The variables matched for PSM included age at diagnosis, gender, ECOG, comorbidity, HBV, ascites, HCC number, HCC diameter, ALBI grade, AFP, tyrosine kinase inhibitors and immunotherapy. uHCC, unresectable hepatocellular carcinoma; SR, surgical resection; MWA, microwave ablation; PSM, propensity score match. .

Crude Kaplan-Meier analyses following PSM revealed no significant differences in cumulative ARFS rates between SR and MWA groups: 97.5%, 90.4%, 84.3%, and 51.7% in the SR group versus 96.8%, 89.8%, 76.2%, and 45.6% in the MWA group (**Figure 4A**, p = 1.000). Cumulative ASRFS rates demonstrated no significant differences between both groups: 99.1%, 99.1%, 98.8%, and 97.6% for the SR group versus 99.0%, 97.7%, 96.3%, and 94.4% for the MWA group (**Figure 4B**, p = 0.250). Additionally, no significant differences were observed in cumulative RFS rates: 100%, 93.2%, 77.2%, and 37.5% in the SR group versus 96.8%, 90.1%, 75.0%, and 27.9% in the MWA group (**Figure 4C**, p = 0.412). Similarly, cumulative OS rates showed no significant differences between both groups: 98.5%, 92.2%, 85.3%, and 67.9% in the SR group versus 98.3%, 91.8%, 82.5%, and 52.3% in the MWA group (**Figure 4D**, p = 0.211).

### Another analytic methods for survival outcomes comparison

**Table 2** presents the comparison of four types of survival outcomes between the two downstaging treatment modalities using different analytic methods. For OS comparison, the unadjusted hazard ratio (HR) was 0.563 (95% confidence interval [CI]: 0.382-0.728; *p* = 0.011), with BMI and serum AFP level identified as independent covariates (both P < 0.05). After adjusted analysis, the HR between the two groups was 0.645 (CI: 0.530-1.758; *p* = 0.404). In the IPTW analysis, the adjusted HR for OS was 0.642 (95% CI: 0.532-1.764; *p* = 0.310). For RFS comparison, the unadjusted HR was 0.650 (95% CI: 0.411-1.822; *p* = 0.592), with the interval between local regional treatment and TACE, and systemic treatment as independent covariates (both *P* < 0.05). SR provided comparable survival benefits to MWA based on unadjusted, adjusted, and IPTW analyses. Similarly, SR and MWA exhibited comparable survival benefits for ARFS and ASRFS across all three analytic methods.

**Table 2:** Predictions of time-dependent endpoints in the SR group versus MWA group according to analytic method.

Analysis	HR*	95% CI	P-value
OS			
Unadjusted	0.563	0.382-0.728	0.011
Adjusteda	0.645	0.530-1.758	0.404
IPTW	0.648	0.532-1.764	0.310
PSM	0.644	0.538-1.676	0.211
RFS			
Unadjusted	0.650	0.411-1.822	0.592
Adjustedb	0.711	0.487-1.269	0.612
IPTW	0.607	0.514-1.777	0.665
PSM	0.612	0.687-1.569	0.413
ARFS			
Unadjusted	0.352	0.211-2.698	0.231
Adjustedc	0.435	0.410-1.098	0.578
IPTW	0.422	0.314-1.268	0.895
PSM	0.312	0.087-1.956	1.000
ASRFS			
Unadjusted	0.650	0.415-1.822	0.592
Adjustedd	0.711	0.487-1.269	0.612
IPTW	0.607	0.514-1.777	0.665
PSM	0.612	0.687-1.569	0.413

* HRs for the SR group compared with the MWA group.

^a^Adjusted for BMI, and serum AFP level, which were significant in the univariate analysis.

^b^Adjusted for the AFP level, HCC_diameter and systemic treatment, which were significant in the univariate analysis.

^c^Adjusted for HBV, and serum AFP level, which were significant in the univariate analysis.

^d^Adjusted for comorbidity, HBV, HCC_diameter, OR to the first TACE, and serum AFP level, which were significant in the univariate analysis. SR, surgical resection; MWA, microwave ablation; PSM: propensity score match; IPTW, inverse probability treatment weighting; HR, hazard ratio; CI, confidence interval;OS, overall survival; RFS, recurrence-free survival; ARFS, aggressive recurrence-free survival;. ASRFS, advanced-stage recurrence-free survival.

### Risk factors for survival outcomes

Both univariate and multivariate analyses were executed to determine risk factors for OS and RFS (**Table 3**). Univariate analysis revealed that Body Mass Index (BMI) (HR: 0.66; 95% CI: 0.42-0.81; *p* = 0.024) and AFP (HR: 2.24; 95% CI: 1.26-3.98; *p* = 0.006) represented significant factors linked to poor OS. Multivariate analysis demonstrated that BMI (HR: 0.61; 95% CI: 0.37-0.82; *p* = 0.006) remained the sole factor markedly influencing OS. Univariate analysis identified AFP (HR: 2.05; 95% CI: 1.06-3.63; *p* = 0.013), tumor size (HR: 1.67; 95% CI: 1.34-2.09; *p* = 0.035), the interval between local regional treatment, including SR and MWA, and TACE (HR: 0.46; 95% CI: 0.26-0.81; *p* = 0.008), and systemic treatment (HR: 0.35; 95% CI: 0.21-0.82; *p* = < 0.001) as significant factors for poor RFS. Multivariate analysis indicated that the interval between local regional treatment, including SR and MWA, and TACE (HR: 0.41; 95% CI: 0.24-0.67; *p* = 0.002), HBV (HR: 0.32; 95% CI: 0.15-0.69; *p* = 0.004) and systemic treatment (HR: 0.44; 95% CI: 0.25-0.76; *p* = 0.001) markedly influenced RFS. Risk factors for ASRFS and ARFS identified by multivariate analysis are presented in **Supplementary Tables 2, 3**.

**Table 3:** Risk Factors Associated with OS, and RFS of patients with HCC after Downstaging according to Univariate and Multivariate Analysis.

Variables	OS		RFS	
	Univariate analysis	Multivariate analysis	Univariate analysis	Multivariate analysis
	HR (95% CI, p value)	HR (95% CI, p value)	HR (95% CI, p value)	HR (95% CI, p value)
Age (years)				
≤65	Ref	Ref	Ref	Ref
>65	1.14(0.54-2.37, p = 0.735)	1.20 (0.54-2.69, p = 0.650)	1.24 (1.00-1.53, p = 0.050)	1.53 (0.67-3.48, p = 0.315)
Sex				
Female	Ref	Ref	Ref	Ref
Male	0.72 (0.40-1.31, p = 0.284)	0.61 (0.31-1.21, p = 0.156)	1.26 (1.00-1.59, p = 0.054)	0.53 (0.27-1.03, p = 0.063)
BMI (kg/m^2^)				
≤23.3	Ref	Ref	Ref	Ref
>23.3	0.66 (0.42-0.81, p = 0.024)	0.61 (0.37-0.82, p = 0.006)	0.58 (0.42-0.89, p = 0.041)	0.73 (0.46-1.07,p = 0.052)
Comorbidity				
Absence	Ref	Ref	Ref	Ref
Presence	1.21 (0.67-2.19, p = 0.518)	1.29 (0.64-2.58, p = 0.472)	1.28 (1.00-1.63, p = 0.048)	0.81 (0.42-1.57, p = 0.529)
HBV				
Absence	Ref	Ref	Ref	Ref
Presence	0.76 (0.37-1.69, p = 0.147)	0.84 (0.46-1.72, p = 0.305)	1.33 (1.03-1.71, p = 0.026)	0.32 (0.15-0.69, p = 0.004)
ALBI grade				
1	Ref	Ref	Ref	Ref
2	1.53 (0.93-2.53, p = 0.097)	1.58 (0.91-2.72, p = 0.102)	1.19 (0.98-1.44, p = 0.082)	1.58 (0.91-2.72, p = 0.102)
HCC_number after TACE				
1	Ref	Ref	Ref	Ref
2-3	1.14 (0.70-1.86, p = 0.584)	1.57 (0.93-2.64, p = 0.690)	1.17 (0.82-1.65, p = 0.388)	1.51 (0.90-2.54, p = 0.118)
HCC_diameter (cm) after TACE				
≤ 3	Ref	Ref	Ref	Ref
> 3	0.78 (0.67-5.87, p = 0.283)	0.90 (0.51-1.59, p = 0.724)	1.67 (1.34-2.09, p = 0.035)	0.98 (0.57-1.68, p = 0.937)
AFP (ng/ml)				
≤ 400	Ref	Ref	Ref	Ref
> 400	2.24 (1.26-3.98, p = 0.006)	1.88 (0.84-3.56, p = 0.332)	2.05 (1.06-3.63, p = 0.013)	1.79 (0.95-3.37, p = 0.069)
The median interval (days)				
≤ 60	Ref	Ref	Ref	Ref
> 60	0.54 (0.40-1.77, p = 0.324)	0.52 (0.44-1.78, p = 0.205)	0.46 (0.26-0.81 p = 0.008)	0.41 (0.24-0.67, p = 0.002)
Systemic treatment				
Absence	Ref	Ref	Ref	Ref
Presence	1.27 (0.88-1.92, p = 0.303)	1.41 (0.83-1.88, p = 0.215)	0.35 (0.21-0.82, p < 0.01)	0.44 (0.25-0.76, p = 0.01)
Treatment modality				
SR	Ref	Ref	Ref	Ref
MWA	0.56 (0.38-0.72, p = 0.011)	0.60 (0.44-1.21, p = 0.211)	0.65 (0.41-1.82, p = 0.592)	0.64 (0.45-1.76, p = 0.465)

Cox regression analyses are used to calculate the hazard ratios (HRs) and 95% confidence intervals (CIs) based on OS and RFS. HCC, hepatocellular carcinoma; SR, surgical resection; MWA, microwave ablation; BMI, body mass index; ECOG, Eastern Cooperative Oncology Group; HBV, hepatitis type B viral; AFP, α-fetoprotein; ALBI, albumin-bilirubin; HR, hazard ratio; CI, confidence interval; OS, overall survival; RFS, recurrence-free survival.

### Subgroup analysis

**Figure 5:**
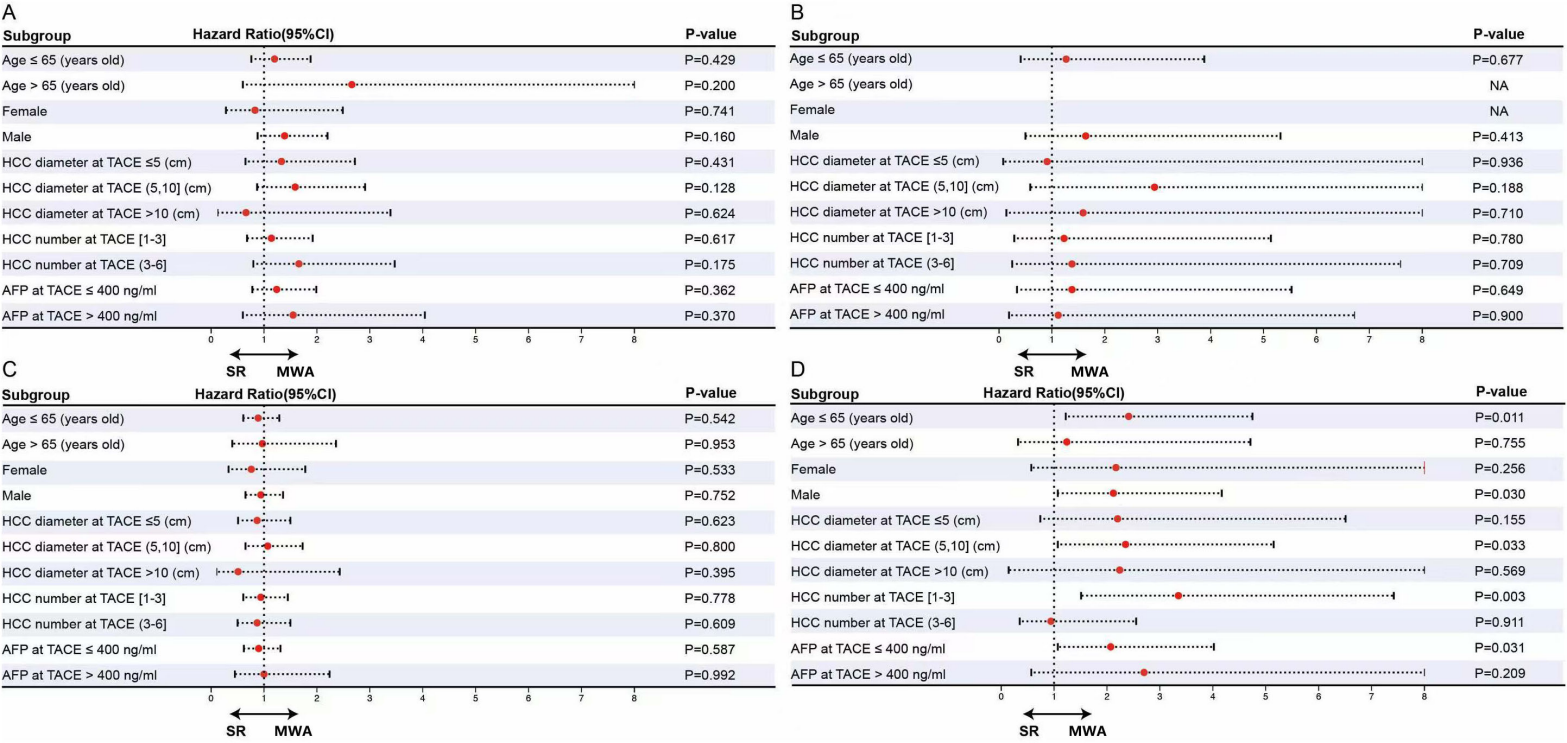
Subgroup analyses of SR and MWA groups for uHCC patients in downstaged cohorts. Forest plots show that the factors associated with **(A)** aggressive recurrence-free survival (ARFS) and **(B)** advanced-stage recurrence-free survival (ASRFS), **(C)** progression-free survival (PFS) and **(D)** overall survival (OS) of uHCC patients between SR group and MWA group.

Subgroup analysis based on several key preoperative variables for the two treatment protocols (SR vs. MWA) is presented as forest plots for survival outcomes (**Figure 5**). Notably, patients with the following characteristics—male gender, tumor diameter of 5–10 cm, 1–3 tumors, and AFP ≤ 400 ng/ml—derived greater OS in the SR group than that in the MWA group.

## Discussion

Previously, downstaging therapy has been defined as the conversion of intermediate-stage tumors to early-stage tumors using interventional treatments such as TACE or transarterial radioembolization (TARE) (19, 20), which enables patients with initially unresectable HCC to receive curative-intent treatment. The 2022 Chinese Expert Consensus on Conversion Therapy for Hepatocellular Carcinoma demonstrates that downstaging therapy provides long-term survival advantages for individuals with intermediate-stage HCC (21). To our knowledge, no research has presently documented comparisons of long-term outcomes between SR and MWA after downstaging for intermediate-stage HCC. This multi-center investigation sought to evaluate the efficacy and long-term outcomes between SR and MWA in HCC patients who achieved downstaging following TACE.

Due to this study’s retrospective design, data collection from seven hospitals was implemented to strengthen real-world heterogeneity relative to single-center approaches. Multiple statistical adjustment techniques were utilized for comparing long-term outcomes between SR and MWA cohorts, seeking to deliver evidence-based support for minimally invasive local regional therapy following TACE. Furthermore, inclusion was limited to patients achieving OR to maintain consistency regarding TACE therapeutic efficacy in intermediate-stage HCC, facilitating balanced baseline characteristics across the two subsequent treatment cohorts.

In this study, we reviewed 26.1% (554/2,125) of intermediate-stage HCC patients who achieved an OR to TACE and received downstaging therapy with a median of 2 sessions, providing evidence for TACE-based downstaging treatment. As a result, the mean maximum tumor diameter decreased from 7.6 ± 1.1 cm to 4.2 ± 0.5 cm after multiple cycles of TACE, indicating that the targeted lesions achieved downstaging and met the Milan criteria. Comparison of long-term outcomes between the downstaging and non-downstaging cohorts revealed that the 10-year cumulative OS and RFS rates were 63.5% and 57.6%, respectively, in the downstaging group, relative to 32.3% and 22.6% in the non-downstaging group. This notable enhancement in long-term survival aligns with earlier research that recognized minimal tumor burden as a beneficial factor for extended survival (22, 23).

With a median follow-up period exceeding 5 years, we compared four types of long-term survival outcomes (OS, RFS, ARFS, ASRFS) between MWA and SR following TACE using four analytic methods. The results can be summarized as follows: (1) SR and MWA exhibited comparable long-term OS and RFS before and after three adjusted analyses, indicating that MWA can achieve similar long-term survival to SR in patients who successfully underwent downstaging treatment; (2) For the two special types of RFS (ARFS and ASRFS), no significant differences were noted between the SR and MWA groups before and after adjusted analyses.

The potential reasons for these findings are as follows: 1) In this study, a single MW antenna was used for lesions < 3 cm, two MW antennas for lesions < 5 cm, and multi-session MWA for lesions > 5 cm, all of which were performed successfully. Furthermore, the ongoing advancement of supportive technologies—encompassing artificial fluid infusion, three-dimensional ablation planning systems, and multi-modal image fusion navigation—along with the synergistic effect of TACE combined with MWA contributes more effectively to the complete inactivation of tumor cells (24). Meanwhile, segmental or hemihepatectomy enables R0 resection achievement, resulting in comparable recurrence outcomes between both groups; 2) When recurrence occurs in patients after downstaging, new treatment strategies are promptly implemented. In addition, the rapid advancement of targeted immunotherapy in recent years (25–27) has resulted in no significant differences in recurrence types between the two groups, thus leading to no significant differences in long-term overall survival. This result is consistent with previous comparisons between MWA and SR in patients with 3–5 cm HCC (16).

Multivariate Cox regression analyses demonstrated that BMI serves as a crucial risk factor for long-term OS, aligning with prior findings. Notably, the median interval between TACE and SR/MWA, along with systemic treatment, serves a pivotal function in enhancing long-term RFS following downstaging therapy for unresectable HCC. The potential reasons are as follows: (1) Iodized oil delivered to liver tumors via the hepatic artery gradually accumulates over time, embolizing surrounding satellite lesions and microvascular invasion. For TACE treatment of intermediate-stage HCC, an interval of more than two months appears to be a reasonable time point for effective embolization; (2) TACE-induced embolization triggers tumor hypoxic necrosis, enhances programmed cell death protein-L1 (PD-L1) inhibitor expression, and subjects tumor cells to severe hypoxic or anaerobic conditions, resulting in free vascular endothelial growth factor (VEGF) release (28). Immune checkpoint inhibitors (ICIs) augment T cell anti-tumor immune responses through PD-L1/PD-1 pathway blockade, whereas molecular targeted therapy—comprising humanized monoclonal antibodies with specific VEGF binding capacity—eliminates circulating VEGF and facilitates tumor microenvironment conversion from immunosuppressive to immune-supportive states (29). Therefore, combining these three methodologies may yield potential synergistic benefits. In addition, comorbidities may increase the risk of postoperative recurrence by altering circulating metabolism in HCC patients.

Subgroup analysis indicated that special populations require careful consideration when choosing post-downstaging treatment, such as male patients, those with tumor sizes of 5–10 cm, 1–3 tumors, and AFP ≤ 400 ng/ml. In these patients, SR provides better disease control than MWA. This finding helps interventional radiologists develop preoperative follow-up treatment plans, thereby improving the survival time of HCC patients.

Despite the strengths of this study—encompassing a substantial cohort and multi-institutional design—several limitations exist. First, although PSM and IPTW were utilized to balance variables, inherent biases remain due to the retrospective study design and sample reduction after PSM. PSM can only adjust for measured confounders. Unmeasured factors (e.g., precise tumor location relative to bile ducts/vessels, patient socioeconomic status, implicit surgeon/radiologist preference) could still significantly influence treatment assignment and outcomes. Second, this investigation included patients from various hospitals throughout China, and variations in TACE protocols and chemotherapy drug administration duration among institutions may have influenced the final survival outcomes. Third, no data were gathered regarding tumor positioning in hilar or subcapsular areas, preventing direct comparison of tumors in these anatomical locations between the two cohorts. Fourth, this study enrolled Chinese patients with predominantly large HCC and HBV infection as the primary etiology. Whether these findings can be broadly extrapolated to Western populations, where most patients exhibit reduced tumor burden or alcoholic liver cirrhosis as the primary etiology, remains uncertain. Moving forward, prospective clinical trials should be implemented to further validate the reliability and reproducibility of these study findings. Fourth, the study did not analyze tumor pathology or molecular subtypes (e.g., histological grade, microvascular invasion, CTNNB1 mutations). These are known to be powerful prognostic factors that could confound the comparison. Fifth, while all patients had Child-Pugh A liver function, the presence and severity of clinically significant portal hypertension (e.g., esophageal varices, splenomegaly, thrombocytopenia) were not evaluated. This is a major predictor of post-hepatectomy outcomes.

In conclusion, MWA may be an acceptable alternative to SR as a first-line therapeutic approach for intermediate-stage HCC patients who achieve an OR to TACE. Our findings demonstrate that TACE-based downstaging therapy is a feasible strategy for intermediate-stage HCC and contributes to improved long-term survival. In particular, MWA technology has achieved substantial advancement in recent years and demonstrates comparable RFS and OS outcomes to SR, making it a viable minimally invasive alternative when SR is technically challenging to implement. Additionally, SR remains the gold standard for suitable candidates, especially those in the subgroups that benefitted more from it. A prospective clinical trial should be designed in the future to validate our findings.

## Data Availability

The datasets presented in this article are not readily available because Data generated or analyzed during the study are available from the corresponding author by request. Requests to access the datasets should be directed to Wendao Liu, Liu_wendao_2023@163.com.
