# Supplementary material for: Downstaging therapy of intermediate-stage hepatocellular carcinoma: microwave ablation vs. surgical resection
Source: Front Oncol. 2026 Aug 5;16:1868497. doi: 10.3389/fonc.2026.1868497 (PMC13485566; doi:10.3389/fonc.2026.1868497)
Supplement: Supplementary file 1 [file DataSheet1.doc]

**Downstaging Therapy of Intermediate-stage Hepatocellular Carcinoma: Microwave Ablation vs. Surgical Resection Running title:** An exploratory study

**This supplementary material includes:**

**1.Supplementary methods and results**

**E 1.1** Indication for TACE downstaging therapy

**E 1.2** TACE procedure

**E 1.3** Criteria for protocol treatment discontinuation

**E 1.4** SR procedures

**E 1.5** MWA procedures

**E 1.6** Clinical data selection

**2.Supplementary tables**

**Table S1.** Post-recurrence treatment in follow-up.

**Table S2.** Prognostic factor analysis for aggressive recurrence-free survival.

**Table S3.** Prognostic factor analysis for advanced-stage recurrence-free survival.

**3.Supplementary figures**

**Figure S1.** Survival curves with log-rank test stratified by MVI without the propensity score matching (PSM) adjusted. (A) Crude overall survival (OS) comparison between MVI-postive group and MVI-negative group; (B) Crude recurrence-free survival (RFS) comparison between MVI-postive group and MVI-negative group. (C) Aggressive recurrence-free survival (ARFS) comparison between MVI-postive group and MVI-negative group. (D) Advanced-stage recurrence-free survival (ASRFS) comparison between MVI-postive group and MVI-negative group.

**Figure S2.** Standardized differences in mean or proportion of variables before and after propensity score matching in patients with hepatocellular carcinoma received transarterial chemoembolization downstaging therapy

**1. Supplementary methods and results**

**E 1.1 Indication for TACE downstaging therapy**

In this study, TACE conversion therapy included sequential SR and MWA. SR or MWA was indicated based on the following criteria: (1) intrahepatic nodules that were treated with HAIC were evaluated as partial response (PR) and stable disease (SD) for at least 8 weeks; (2) an R0 resection had to be achieved with a sufficient remnant liver volume (≥ 40% of the standard liver volume for the patients with liver cirrhosis or ≥ 30% of the standard liver volume for the patients without liver cirrhosis); (3) normal coagulation disorders (i.e., prothrombin time < 15 s, prothrombin activity > 40%, and platelet count > 50 cells×10^9^/L); (4) no severe or persistent adverse events (AEs) from HAIC; and (5) Patients with serious comorbidities, including heart, lung and renal dysfunction, etc.

The target tumors were downstaged to BCLC-A stage from BCLC-B stages and the tumor burden was reduced met the Milan criterion after multi-cycle of HAIC treatment. The diameter of large HCC in BCLC-A stage (diameter > 5 cm) was reduced to < 5 cm. The downstaged HCC patients were conducted by surgical resection, and MWA. Among them, resectable tumor was defined as the complete removal of all macroscopic tumor tissue and an expected remnant liver volume no less than 250 mL/M. Complete ablation was defined as the absence of enhancement of any areas of the mass at a follow-up contrast-enhanced imaging examination performed 1 month after MWA.

**E1.2 TACE procedure**

TACE procedure has been described in our previous report [1, 2]. All equipment of IAT procedures included i) digital subtraction angiography (Philips, type FD 20 1250 mA, Amsterdam, Netherlands); ii) the artery sheath catheter was inserted into the femoral artery using the modified Seldinger technique; iii) A 5-Fr Yashiro catheter (Terumo, Tokyo, Japan) was advanced into the celiac trunk and superior mesenteric artery to assess the feeding hepatic artery; iv) A 2.7-Fr micro-catheter (Terumo, Tokyo, Japan) was inserted in the feeding artery. The feeding artery was selected or super-selected whenever possible. Emulsion, which consisted of 10–20 ml lipiodol, 30–50 mg platinum drugs, and 20–40-mg epirubicin was injected slowly until the offending vessel occluded. If necessary, embolization using gel foam mixed with contrast medium was injected to reduce the residual blood flow until there was no longer any tumor staining after repeat angiography.

**E1.3 Criteria for protocol treatment discontinuation**

A) Tumor progression. The progression disease (PD) were assessed by dynamic CT or MRI based on modified Response Evaluation Criteria in Solid Tumor (mRECIST).
B) Intolerable adverse event

i) Patient cannot resume TACE after 30 days of interruption due to an adverse event;

ii) An adverse event that meets the criteria for TACE dose reduction occurs after the dose was already reduced to the lowest level;

iii) Life-threatening adverse event;

C)The need for another anticancer treatment due to downstaging at the physician’ s discretion;

D) TACE becomes technically infeasible;

E) Inadequate blood or bone marrow (leukopenia count < 3.0×10^9^/L, platelet count < 50×10^9^/L, and hemoglobin < 8.0 g/L)

F) Patient requests to discontinue the study；
G) Death.

**E 1.4 SR procedure**

SR was performed by an open approach under general anesthesia. ‎Two surgeons with 15 to 10 years of experience in hepatectomy.‎ Intraoperative ultrasonography was used routinely to evaluate other possible tumors ‎and the possibility of a negative resection margin. Anatomical resections according to segmentectomy or subsegmentectomy described by Makuuchi et al. [3]were preferred in the surgeries. The resected specimens were histopathologically examined. According to the guidelines of the International Union Against Cancer (UICC), R0 resections‎was defined by the absence (tumor-free margin no less than 1 mm for all detected lesions). When examined under a microscope, there is no cancer cell left at the margins (edges of surgical removal)[4].‎

**E 1.5 MWA procedure**

The microwave unit (KY-2000; Kangyou Medical, Nanjing, China) was capable of producing 100 W of power at 2,450 MHz. The cool tip needle antenna had a diameter of 1.9 mm (15 gauge) and a length of 18 cm. MWA procedure was performed with 16 multidetector-row CT (MDCT) scanner (Brilliance CT BigBore; Phillip Medical Systems, the Netherlands) guidance. Ablation was performed by three interventional radiologists. The antenna or electrode was located into the tumor, and the deployment degree scale was determined according to the tumor size and shape. Patients laid either in a supine or prone position on the scanning bed according to the location of the lesions. Each TA procedure was performed under local and intravenous anaesthesia. After anaesthesia, a 15-gauge, 18-cm antenna (MTC-3C, Nanjing Qinghai Research Institute of Microwave Electric, China) was inserted into the tumor at a predetermined angle. To ensure the appropriate position of the ablation electrode, CT image scanning was performed again before the ablation surgery. Power settings and ablation times were determined according to the standard recommendations provided by the manufacture of the equipment. Each TA session used an overlapping technique to ensure the eradication of the entire tumor.

**E1.6 Clinical data selection**

18 clinical variables are collected as follows: (1) demographic and history variables (ECOG, age, gender, comorbidities (i.e., hypertension, diabetes, heart disease, renal disease a esophageal gastric varices, etc.), etiology, ALBI grade, ascites); (2) tumor features (maximal tumor diameter, number of tumor); (3) laboratory findings (α-fetoprotein [AFP], serum albumin; [ALB], serum total bilirubin [TB], platelet counts, prothrombin time (PT), international normalized ratio [INR], aspartate aminotransferase [AST] and alanine aminotransferase [ALT]). Albumin- bilirubin (ALBI) grades were used to replace CTP grade for their objectiveness. ALBI score was calculated before treatment using the appropriate clinical parameters and ALBI grade was defined as follows: (log 10 bilirubin [BI] [μ mol /L] × 0.66) + (albumin [AL] [g/L] ×-0.085), (grade 1, 2, and 3 = ≤ -2.60, > -2.60 to -1.39, and > -1.39, respectively) [5-6]. TACE combined with targeted immunotherapy. During TACE treatment, molecular-targeted agents (MTAs) and immune checkpoint inhibitors (ICIs) were performed to control the intrahepatic and extrahepatic progression. Oral first-line targeted chemotherapy including sorafenib and lenvatinib was started 1–5 days after the first TACE session and continually administered. Once the disease progresses or 3-4 AEs occur, the second-line treatment regimen (regorafenib or apatinib) can be administered. Oral lenvatinib (Lenvimafi; Eisai Co., Ltd.) was administered to the patients with advanced HCCs. The initial dose was determined based on the patient’s body weight and liver function. Patients weighing > 60 kg with the Child–Pugh A classification started at a dose of 12 mg once daily. Patients weighing < 60 kg with the same liver function started at a dose of 8 mg once daily. A reduction in dosage or interruption of treatment was implemented when AEs were detected. Lenvatinib was administered unless patients were intolerant of radiological tumor progression or AEs. immunotherapy was performed after 1-3 days TACE treat and every 3 weeks intravenously. Fixed-dose administration of PD-1 was used until disease progression or unexpected toxicity. The dose and interval of MAT allowed changes depending on toxicity and disease conditions.

**References**

[1]	Matsui O, Miyayama S, Sanada J, Kobayashi S, Khoda W, Minami T, et al. Interventional oncology: new options for interstitial treatments and intravascular approaches: superselective TACE using iodized oil for HCC: rationale, technique and outcome. J Hepatobiliary Pancreat Sci 2010; 17:407-409.

[2]	Golfieri R, Cappelli A, Cucchetti A, Piscaglia F, Carpenzano M, Peri E, et al. Efficacy of selective transarterial chemoembolization in inducing tumor necrosis in small (<5 cm) hepatocellular carcinomas. Hepatology 2011; 53:1580-1589.

[3] Takeshi Takamoto, Masatoshi Makuuchi. Precision surgery for primary liver cancer. Cancer Biol Med. 2019 8;16(3):475-485.
[4] Kensuke Yamamura, Toru Beppu. Makuuchi's criteria for liver resection in the modern era of functional liver remnant volume evaluation. Hepatol Res. 2023 Feb;53(2):91-92.
[5] Kao WY, Su CW, Chiou YY, et al. Hepatocellular Carcinoma: Nomograms Based on the Albumin-Bilirubin Grade to Assess the Outcomes of Radiofrequency Ablation. Radiology 2017;285:670-680.
[6] Ni JY, Fang ZT, An C, et al. Comparison of albumin-bilirubin grade, platelet-albumin-bilirubin grade and Child-Turcotte-Pugh class for prediction of survival in patients with large hepatocellular carcinoma after transarterial chemoembolization combined with microwave ablation. Int J Hyperthermia 2019;36:841-853.

**2.Supplementary tables**

**Table S1.** Post-study treatment in follow-up.

| **Post-recurrence treatment** | **SR group**  **n=207** | **MWA group**  **n= 347** | **P-Value** |
| --- | --- | --- | --- |
| **Number of patients with at least one 1-line treatment after disease progression** | | | |
| **Total** | 156 (71.2) | 255 (73.5) | 0.824 |
| Thermal ablation | 78 (35.6) | 203 (58.5) |  |
| Surgical resection | 2 (0.9) | 14 (3.9) |  |
| Radiotherapy for vascular invasion | 31 (14.2) | 40 (11.5) |  |
| TACE or HAIC | 67 (28.5) | 100 (28.7) |  |
| TKIs | 89 (40.6) | 99 (28.6) |  |
| ICIs | 47 (24.0) | 77 (22.3) |  |
| **Number of patients with at least one 2-line treatment after disease progression** | | | |
| **Total** | 65 (29.7) | 94 (27.2) | 0.563 |
| Thermal ablation | 6 (2.7) | 25 (6.4) |  |
| Surgical resection | 0 (0) | 0 (0) |  |
| Radiotherapy for vascular invasion | 12 (5.5) | 3 (0.8) |  |
| TACE or HAIC | 45 (23.7) | 92 (26.4) |  |
| TKIs | 27 (12.3) | 52 (14.9) |  |
| ICIs | 19 (8.8) | 28 (8.2) |  |
|  | | | |

**Abbreviation:** TACE, transarterial chemoembolization; HAIC, hepatic arterial infusion chemotherapy; ICIs, immune checkpoint inhibitors; TKIs, tyrosine kinase inhibitors; HCC, hepatocellular carcinoma; SR, surgical resection; TA, thermal ablation.

**Table S2.** Prognostic factor analysis for aggressive recurrence-free survival

| **Variables** | **Aggressive recurrence-free survival** | |
| --- | --- | --- |
|  | **Univariate analysis** | **Multivariate analysis** |
|  | **HR (95% CI, p value)** | **HR (95% CI, p value)** |
| **Age (years)** |  |  |
| ≤65 | Ref |  |
| >65 | 0.99 (0.57-1.73, p=.982) |  |
| **Sex** |  |  |
| Female | Ref |  |
| Male | 1.02 (0.57-1.84, p=.940) |  |
| **BMI (kg/m^2^)** |  |  |
| ≤23.3 | Ref | Ref |
| >23.3 | 0.67 (0.46-0.95, p=.027) | 0.66 (0.45-0.96, p=.031) |
| **Comorbidity** |  |  |
| Absence | Ref | Ref |
| Presence | 2.30 (1.44-3.69, p<.001) | 2.05 (1.16-3.62, p=.014) |
| **Ascites** |  |  |
| Absence | Ref |  |
| Presence | 1.32 (0.18-9.54, p=.780) |  |
| **ALBI grade** |  |  |
| 1 | Ref |  |
| 2 | 0.95 (0.63-1.44, p=.813) |  |
| **HCC_number** |  |  |
| (1-3] | Ref |  |
| (3-6) | 1.07 (0.70-1.62, p=.755) |  |
| **HCC_diameter at TACE** |  |  |
| <=5 cm | Ref |  |
| (5-10] cm | 1.11 (0.72-1.69, p=.642) |  |
| >10 cm | 1.91 (0.89-4.08, p=.097) |  |
| **AFP (ng/ml)** |  |  |
| ≤ 400 | Ref |  |
| > 400 | 1.10 (0.67-1.80, p=.716) |  |
| **Systemic treatment** |  |  |
| Absence | Ref |  |
| Presence | 0.25 (0.17-0.38, p<.001) |  |
| **Treatment modality** |  |  |
| SR | Ref | Ref |
| MWA | 1.29 (0.85-1.97, p=.235) | 0.31 (0.19-0.51, p<.001) |

**Note**: A Cox proportional hazards regression model for extrahepatic progression-free survival was used in all cohort. All variables were included in a multivariate stepwise Cox regression analysis.

**Abbreviation:** HR, hazard ratio; CI, confidence intervals; ECOG, Eastern Cooperative Oncology Group; HBV, hepatitis B virus; ALBI, albumin-bilirubin‎; HAIC, hepatic arterial infusion chemotherapy; AFP: α-fetoprotein; TACE, transarterial chemoembolization; HAIC, hepatic arterial infusion chemotherapy; ICIs, immune checkpoint inhibitors; TKIs, tyrosine kinase inhibitors; HCC, hepatocellular carcinoma; SR, surgical resection; TA, thermal ablation.

**Table S3.** Prognostic factor analysis for advanced-stage recurrence-free survival

| **Variables** | **Advanced-stage recurrence-free survival** | |
| --- | --- | --- |
|  | **Univariate analysis** | **Multivariate analysis** |
|  | **HR (95% CI, p value)** | **HR (95% CI, p value)** |
| **Age (years)** |  |  |
| ≤65 | Ref |  |
| >65 | 0.39 (0.05-3.00, p=.367) |  |
| **Sex** |  |  |
| Female | Ref |  |
| Male | 2.04 (0.27-15.62, p=.491) |  |
| **BMI (kg/m^2^)** |  |  |
| ≤23.3 | Ref | Ref |
| >23.3 | 0.56 (0.23-1.40, p=.218) | 0.49 (0.28-0.83, p=.009) |
| **Comorbidity** |  |  |
| Absence | Ref |  |
| Presence | 0.54 (0.07-4.13, p=.553) |  |
| **ALBI grade** |  |  |
| 1 | Ref |  |
| 2 | 0.69 (0.22-2.14, p=.515) |  |
| **HCC_number** |  |  |
| (1-3] | Ref |  |
| (4-6) | 1.32 (0.46-3.82, p=.606) |  |
| **HCC_diameter at TACE** |  |  |
| <5 cm | Ref | Ref |
| (5-10] cm | 2.74 (0.73-10.34, p=.136) | 2.28 (0.60-8.63, p=.225) |
| >10 cm | 8.85 (1.78-44.01, p=.008) | 7.25 (1.46-36.09, p=.016) |
| **AFP (ng/ml)** |  |  |
| ≤ 400 | Ref |  |
| > 400 | 2.06 (0.69-6.16, p=.195) |  |
| **Systemic treatment** |  |  |
| Absence | Ref |  |
| Presence | 0.11 (0.04-0.33, p<.001) |  |
| **Treatment modality** |  |  |
| SR | Ref | Ref |
| MWA | 1.30 (0.44-3.89, p=.636) | 2.01 (0.95-4.25, p=.067) |

**Note**: A Cox proportional hazards regression model for postoperative progression survival was used in all cohort. All variables were included in a multivariate stepwise Cox regression analysis.

**Abbreviation:** HR, hazard ratio; CI, confidence intervals; ECOG, Eastern Cooperative Oncology Group; HBV, hepatitis B virus; ALBI, albumin-bilirubin‎; HAIC, hepatic arterial infusion chemotherapy; AFP: α-fetoprotein; TACE, transarterial chemoembolization; HAIC, hepatic arterial infusion chemotherapy; ICIs, immune checkpoint inhibitors; TKIs, tyrosine kinase inhibitors; HCC, hepatocellular carcinoma; SR, surgical resection; TA, thermal ablation.

3. **Supplementary figure**

**Figure S1** Survival curves with log-rank test stratified by downstaging without the propensity score matching (PSM) adjusted. (A) aggressive recurrence-free survival (ARFS) and (B) advanced-stage recurrence-free survival (ASRFS), (C) progression-free survival (PFS) and (D) overall survival (OS) of uHCC patients between SR group and MWA group. Survival curves with log-rank test stratified by downstaging with the propensity score matching (PSM) adjusted. (E) aggressive recurrence-free survival (ARFS) and (F) advanced-stage recurrence-free survival (ASRFS), (G) progression-free survival (PFS) and (H) overall survival (OS) of uHCC patients between SR group and MWA group.

**Abbreviations:** uHCC, unresectable hepatocellular carcinoma; SR, surgical resection; MWA, microwave ablation

**Figure S2.** Standardized differences in mean or proportion of variables before and after propensity score matching in patients with hepatocellular carcinoma received transarterial chemoembolization downstaging therapy.
